# Shared Pattern of Endocranial Shape Asymmetries among Great Apes, Anatomically Modern Humans, and Fossil Hominins

**DOI:** 10.1371/journal.pone.0029581

**Published:** 2012-01-05

**Authors:** Antoine Balzeau, Emmanuel Gilissen, Dominique Grimaud-Hervé

**Affiliations:** 1 CNRS, UMR 7194, Département de Préhistoire, Muséum national d'histoire naturelle, Paris, France; 2 Department of African Zoology, Royal Museum for Central Africa, Tervuren, Belgium; 3 Laboratory of Histology and Neuropathology, Université Libre de Bruxelles, Brussels, Belgium; 4 Department of Anthropology, University of Arkansas, Fayetteville, United States of America; Durham University, United Kingdom

## Abstract

Anatomical asymmetries of the human brain are a topic of major interest because of their link with handedness and cognitive functions. Their emergence and occurrence have been extensively explored in human fossil records to document the evolution of brain capacities and behaviour. We quantified for the first time antero-posterior endocranial shape asymmetries in large samples of great apes, modern humans and fossil hominins through analysis of “virtual” 3D models of skull and endocranial cavity and we statistically test for departures from symmetry. Once based on continuous variables, we show that the analysis of these brain asymmetries gives original results that build upon previous analysis based on discrete traits. In particular, it emerges that the degree of petalial asymmetries differs between great apes and hominins without modification of their pattern. We indeed demonstrate the presence of shape asymmetries in great apes, with a pattern similar to modern humans but with a lower variation and a lower degree of fluctuating asymmetry. More importantly, variations in the position of the frontal and occipital poles on the right and left hemispheres would be expected to show some degree of antisymmetry when population distribution is considered, but the observed pattern of variation among the samples is related to fluctuating asymmetry for most of the components of the petalias. Moreover, the presence of a common pattern of significant directional asymmetry for two components of the petalias in hominids implicates that the observed traits were probably inherited from the last common ancestor of extant African great apes and *Homo sapiens*.

These results also have important implications for the possible relationships between endocranial shape asymmetries and functional capacities in hominins. It emphasizes the uncoupling between lateralized activities, some of them well probably distinctive to *Homo*, and large-scale cerebral lateralization itself, which is not unique to *Homo*.

## Introduction

Human brain asymmetries have been documented since the time of Dax [1], [2] and Broca [3] and have been widely investigated for their functional, physiological or behavioural implications. It emerges from several studies that the combination of right frontal and left occipital protrusions represents brain shape asymmetries that are characteristic of the hominin lineage. This combination is usually described as the “torque” pattern and represents the extension of one cerebral hemisphere beyond the other. The larger frontal or caudal projection (petalia or protrusion) is usually coupled with another structural component, a larger lateral extent of the more projecting hemisphere relative to the other (lobar asymmetries). It is currently accepted that this pattern of asymmetries appeared with early *Homo* [4]–[7] and is most common in human right-handed individuals [8]–[14]. These asymmetries were a topic in non human primate brain studies [6], [8], [10], [15]–[20] and raised a special interest in paleoanthropology [4]–[7], [21]–[22] because of their relationships with handedness and, more generally, with specific aspects of human cognition.

The evolutionary advantage of an asymmetrical brain seems to be the enhancement of neural capacity by allowing parallel and separate processing in the hemispheres [23]. More precisely, split-brain studies in humans indeed have revealed that each cerebral hemisphere has its own set of specialized capacities. The left hemisphere is specialized for language and speech and possesses capacities for problem solving that are crucial for human specific behavior. It is also the hemisphere where uniquely human processes aimed at interpreting behavior and at constructing relationships between perceived events and feelings take place. The right hemisphere has its own specializations, such as facial recognition or attentional monitoring but does not possess the overall cognitive capabilities of the left hemisphere. As such, it reacts more directly to perceptual information. The right hemisphere, with its essential role in more general process, such as integration tasks, therefore appears to be another brain system than the left one, which is the leading hemisphere for highly demanding but specific process, such as language and motor actions. Although of similar size and constituted of roughly the same number of neurons, the right and the left hemispheres are not capable of the same higher order cognitive processes (for review, see [24]–[29]). These neuropsychological observations are in concordance with the results of the diffusion-weighted magnetic resonance imaging study of Iturria-Medina [30] that shows, in both human and macaque, that the connectivity system of left and right hemispheres are different, most probably in relationships with their different cognitive functions. Hence, size or neuron number alone cannot entirely explain human intelligence. The study of brain structural asymmetries as anatomical substrate of functional asymmetries in extant humans, great apes, and fossil hominins is therefore of major importance to understand the structural basis of modern human cognition.

The number of brain structural asymmetries observable on endocranial casts and therefore in fossils is however limited. Fortunately, asymmetries of the shape of the brain, which are visible on endocranial casts, are among the most consistent features available for cross-taxa studies on large samples. Still, studies of brain surface asymmetries are complex because of the difficulty to define surface structural parameters and their homologues. The term “petalia” for instance originally refers to the protrusions of one hemisphere beyond the other [31] and is employed here in this sense although it is now widely used in reference to both protrusions and lobar asymmetries or to cerebral asymmetries revealed by voxel-based morphometry [4]–[8], [19]–[21], [32]–[34]. Moreover, it is possible that gross anatomic asymmetries reflect combined asymmetries in brain subregions. Quantification of surface or volume of these discrete anatomical areas may therefore be biased if their pattern of asymmetry is defined in reference to gross anatomical brain parts.

Be that as it may, endocranial casts accurately reflect brain shape [35] and are the only available material to study fossil cerebral anatomy. Nevertheless, only one study [6] considered the pattern of fronto-occipital protrusions in great apes and fossil hominins from a qualitative viewpoint on the basis of large samples and none supplied quantitative data for these features.

In this context, we developed a methodology for quantifying the various components of endocranial petalias (the antero-posterior, vertical and lateral components of the protrusions of the frontal and occipital poles) (method and 35). The sample of extant specimens and the fossil record studied here is the largest ever used to analyse this feature, both for the number of specimens and for the number of species. Moreover, this reproducible methodology will allow comparisons between studies in the future. We test the possible variation for the pattern of antero-posterior endocranial shape asymmetries between anatomically modern humans (AMH) and African great apes (bonobos, chimpanzees and gorillas, abbreviation GA). We also explore the distribution of these features among large samples of fossil hominins. Finally, we discuss the implications of our results in terms of relationships between endocranial shape asymmetries and functional or behavioural capacities in hominins.

## Results

**Figure 1:**
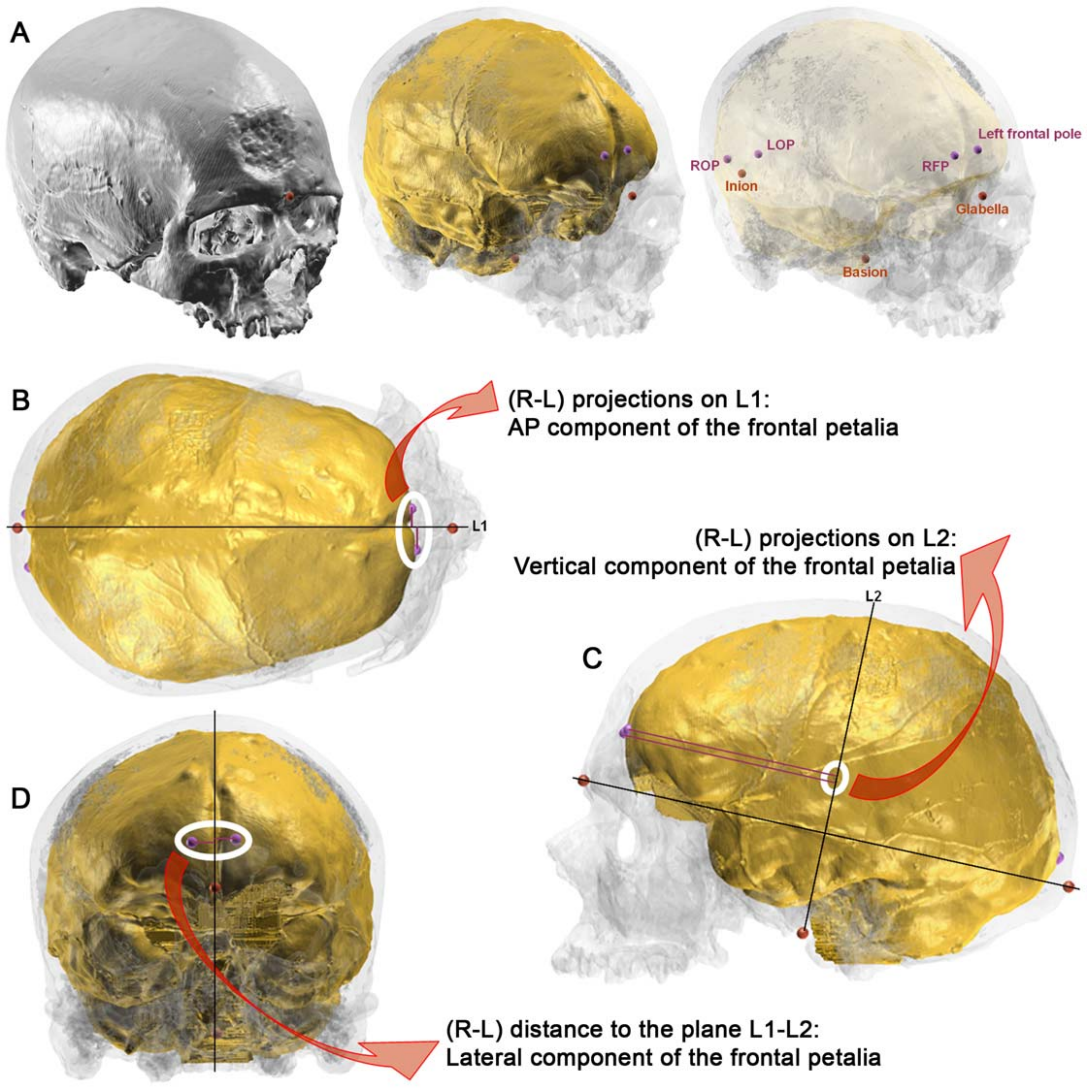
Illustration of the protocol used to quantify the endocranial petalias. A: 3D models of the skull (grey) and of the virtual endocast (yellow) of Cro-Magnon 1 displayed at different levels of transparency. 3 landmarks are positioned on the skull (G: glabella, B: basion, I: inion) and 4 on the endocranial surface (RFP, LFP: right and left frontal poles, ROP, LOP: right and left occipital poles). B: superior view showing a line (L1) traced through glabella and inion. Frontal and occipital poles are orthogonally projected on this line. The distance between the projected images of the points corresponds to the antero-posterior component of petalia (see details for the frontal poles). C: lateral view showing a second line (L2) traced through basion orthogonally to the first line. The 4 endocranial points are projected orthogonally on L2 and the distance between the projected images of the points corresponds to the vertical component of petalia. D: the 4 points are projected orthogonally on the plane defined by the two lines and the difference between the right and left side for this lateral distance constitutes the lateral component of petalia.

Our objective was to determine the variation in location of the most protruding points on the right and left frontal and occipital lobes (*i.e.* the frontal and occipital poles). We defined an external and independent referential based on anatomical points on the skull (glabella, inion and basion; Fig1A). By using this procedure [35], the quantification of endocranial asymmetries was unbiased because we used a reference system, independent of the endocast itself. Moreover, we quantified the difference in location between pairs of relevant landmarks rather than the absolute value of the metrical traits on both sides. Moreover, in order to precisely describe the pattern of variation of endocranial petalias (protrusions), we dissociated the different components (antero-posterior, vertical and lateral) of their spatial location by determining which one of the right or left most protruding point on the frontal or occipital lobes is located more anteriorly or posteriorly than the other (Fig. 1B), which one is located above the other (Fig. 1C) and which one has a more lateral position (Fig. 1D). Positive values corresponded to a right asymmetry and negative values indicated a left asymmetry. A positive value therefore indicated a right point, which was more anterior, lateral or superior to the left point for the different components of the petalias. A negative value indicated a left point, which is more posterior to the right point for the antero-posterior component of the occipital petalia. Different classical terms and indices were used to describe and to identify departures from symmetry [35]–[40]. Subtle departures from symmetry are described by frequency distributions of Right-Left (R-L). Signed asymmetry is the difference between the right and left side for each petalia in an individual (R*_xi_*-L*_xi_*). It retains information about the direction of the asymmetry. Absolute asymmetry is the absolute value of the difference between the right and left side for each petalia in an individual (|R*_xi_*-L*_xi_*|). It also corresponds to FA1 [36]. FA4a estimates the variability of one trait within a given sample, and is calculated using the formula 0.798√var(R*_xi_*-L*_xi_*). These two indices are biased when directional asymmetry or antisymmetry are present [37]. The parameter FA11 quantifies the asymmetries for all individuals and for all traits, and is calculated using the formula Σ(Σ|R*_xi_*-L*_xi_*|)/N [35], [37], [39]. This parameter is cumulative and is useful for comparisons of the global size of asymmetry between samples.

Fluctuating asymmetry (FA) is a pattern of bilateral variation in a sample where the mean of (R-L) is zero and variation is normally distributed about that mean [37]. Directional asymmetry (DA) is a pattern of bilateral variation in a sample that occurs when a side is statistically larger than the other side. DA is detected by statistical tests for departures of the mean (R-L) from zero [35]. Antisymmetry is a pattern of bilateral variation in a sample that occurs when a significant difference exists between sides, but when the larger side varies randomly among individuals [37]. Antisymmetry is detected by statistical tests for departures of frequency distributions of (R-L) from normality in the direction of platykurtosis [37]. FA, DA and antisymmetry are the three main patterns of subtle departures from symmetry exhibited by a sample of individuals [38], [40].

### Do size and gender matter?

We first examined the possible relationships between variation and absolute size (|R*_xi_*-L*_xi_*|) of petalial components, endocranial volume (EV), and sexual dimorphism in the various subsamples. In *Homo sapiens* and in hominins, only the lateral component of the frontal petalia is significantly correlated (p<0.05) with EV (represented by the cube root of the endocranial volume). The correlation is however significant for all the analysed variables when the whole hominid sample is considered (non-parametric and parametric tests, Table S1). This trend among hominids has a major influence on analyses of endocranial asymmetries because endocranial volume rises by a factor of 4 between great apes and recent hominins. Concerning putative gender-related variation, a common non significant pattern is observed in bonobos, chimpanzees and gorillas [35]. Similarly, the means for males and females are not different in extant *Homo sapiens* (Hotelling's T-squared, p = 0.827). Based on these results, we used relative metric data (*x_i_*/^3^√EV_i_*100) to analyse morphological variation of the different components of the petalias and pooled samples with known sexual attribution.

### Testing for asymmetries, their pattern in AMH and GA

Absolute values for FA11 are 19.2 mm for anatomically modern humans (AMH), 15 mm for fossil hominins and 9 mm for extant African great apes (GA). The relative value for FA11 with size correction (*x_i_*/^3^√EV_i_*100) is 17 for AMH, 15.2 for fossil hominins and 12.8 for GA. Means |R-L| vary in the order lateral>vertical>antero-posterior components for both frontal and occipital petalias in AMH. The same order is observed for the occipital petalia in GA and fossil hominins whereas it follows the order vertical>lateral>antero-posterior for the frontal petalia. In addition, when the whole multivariate dataset is considered, extant and fossil AMH are not different (Hotelling's T-squared, p = 0.237), neither are AMH (extant and fossil) and fossil hominins (p = 0.155). Great apes however are significantly different from the whole hominin sample (AMH and fossil hominins) (p<0.01).

**Figure 2:**
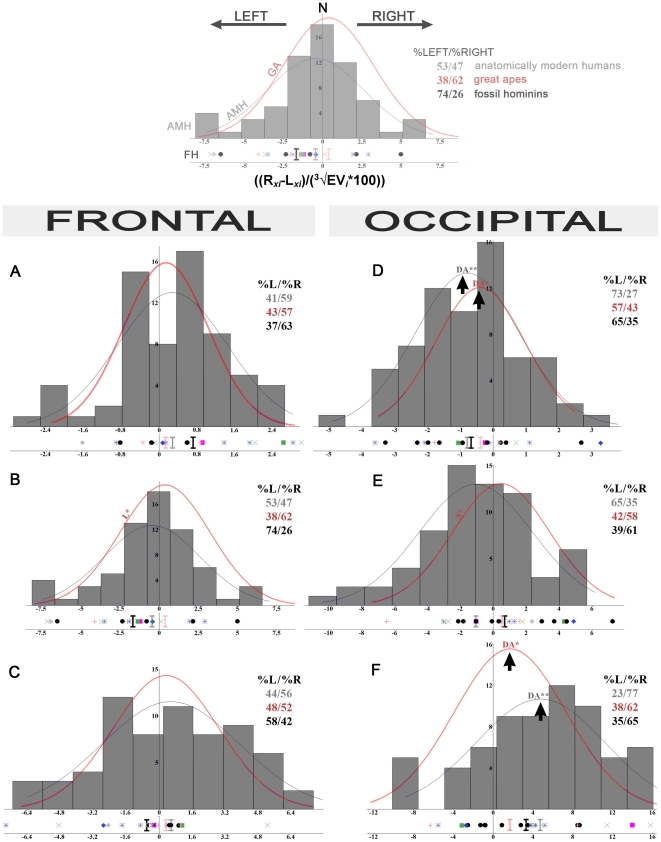
Frequency distribution of petalia components in hominids. Frequency distribution (y axis; N: number of individuals for each bin) of size-corrected (R-L) antero-posterior (A and D), vertical (B and E) and lateral (C and F) components of the frontal (A–C) and occipital (D-F) petalias (x axis: ((R*_xi_*-L*_xi_*)/(^3^√EV_i_*100))) in anatomically modern humans (AMH: grey histograms), curves of fitted normal distributions (parametric estimation) for AMH (grey) and great apes (GA, red). DA indicates significant directional asymmetry (highlighted by a black arrow), L leptokurtosis and S skewness, * indicates a p value <0.05, **<0.01 after Bonferroni correction for multiple tests. %L/%R: lateral distribution of each component of the petalias in AMH (values in grey), great apes (red) and fossil hominins (black). Below the graphs are indicated individual values for fossil hominins (FH): grey diamond: Sts 5, pink square: KNM-WT 17000, green square: KNM-ER 1813, green crosses: KNM-ER 3733, 3883, OH 9, red crosses: Broken Hill, LH 18, blue stars: Ngandong 1, 7, 12, Ngawi, Sambungmacan 3, blue diamond: Liang Bua 1, black dots: Petralona, Gibraltar, Guattari, La Chapelle-aux-Saints 1, Saccopastore 1 and (only for the occipital petalia) La Ferrassie 1, La Quina H5, Spy 1, 10. Black vertical line: mean value for fossil hominins, grey: mean AMH, red: mean great apes.

Mean values of the size-corrected classical indices used to describe asymmetries ((R-L), |R-L| and Fa4a, Table S2, [37]) in AMH are always larger than in GA for the 3 components of both the frontal and occipital petalias. More precisely, these indices show a rightward asymmetry for the antero-posterior (AP) and lateral component of the frontal petalia in AMH and in GA. The AP and lateral component of the occipital petalia also show similar directional asymmetries in both samples, either leftward or rightward. In contrast, the vertical component of the frontal and occipital petalias shows a leftward asymmetry in AMH. These observations are directly related to the values and orientation of the lateral distribution of the petalia components, with more lateralised distribution in AMH (except for the vertical component of the frontal petalia, Table S3). Nevertheless, an important observation is that mean (R-L) is never larger than FA4a [36], illustrating that the distribution of each of the component of the petalias is only slightly asymmetric in AMH and GA samples (Table S2). Most of the distributions of petalial components are close to normal distribution. Only the vertical component of the frontal petalia of GA is significant for kurtosis and the vertical component of the occipital petalia of AMH is significant for skewness (Fig. 2). More importantly, the frequency distribution of the size-corrected (*x_i_*/^3^√EV_i_*100) dimensions (R-L) of the petalial components (antero-posterior, vertical and lateral) illustrates (Fig. 2) the presence of another kind of asymmetry in both AMH and GA for two components of the petalias. Directional asymmetry (DA) is a pattern of bilateral variation that occurs when one side is statistically larger than the other side in a given sample population. DA is detected by statistical tests for departures of the mean (R-L) from zero [38]. Significant DA is present for the antero-posterior (AP) component of the occipital petalia in favor of the left side, and for its lateral component in favor of the right side in AMH (p<0.01) but also in GA (p<0.05). These significant asymmetries are associated with prevalence of individuals with an asymmetry toward the corresponding side, respectively 73%R and 77%L in AMH, 57%R and 62%L in GA (Table S3). Other components of the petalias are not significantly asymmetric, neither in AMH nor in GA, and the observed variation for these features is therefore related to fluctuating asymmetry.

**Figure 3:**
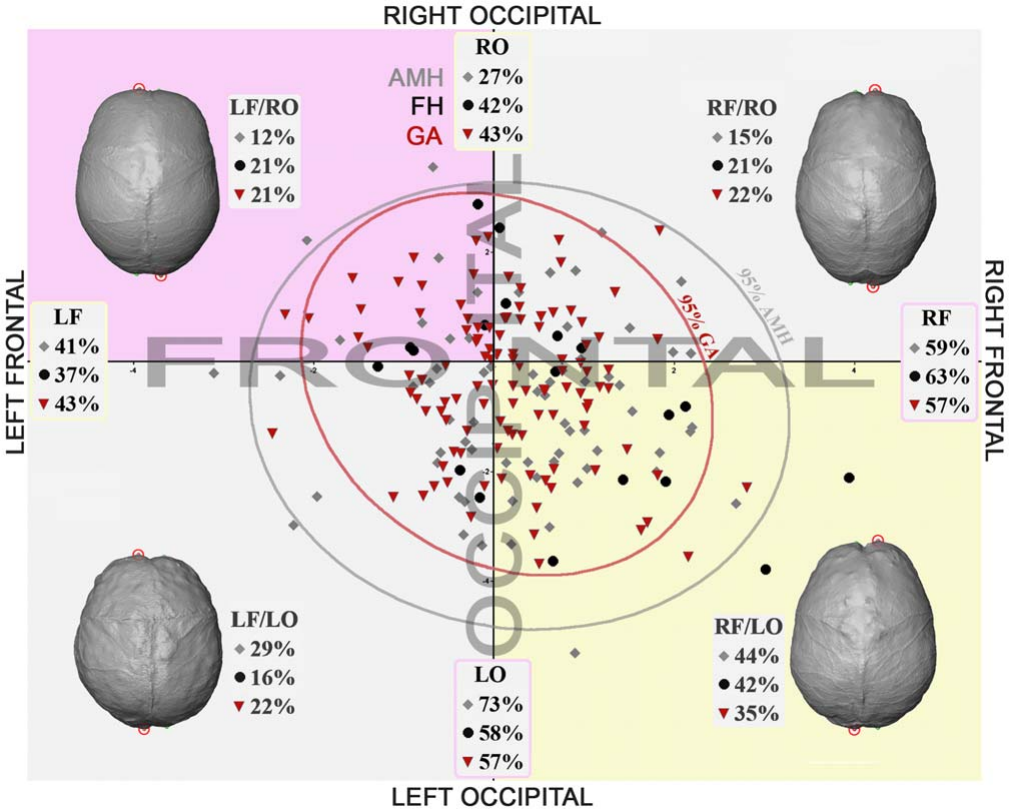
Biplot of AP petalia components. Biplot of the antero-posterior (AP) component of the frontal (x axis) and occipital (y axis) petalias in anatomically modern humans (AMH, grey diamonds), fossil hominins (FH, black dots) and great apes (GA, inverted red triangles). Positive value indicates a rightward asymmetry and negative value a leftward asymmetry. Percentages of right and left frontal (RF, LF) and occipital (RO, LO) petalias, illustration and percentages of the different combination of fronto-occipital petalias (RF/LO, LF/RO, RF/RO, LF/LO) for each sample.

We also analysed petalias as non-metric traits. Concerning the patterns of fronto-occipital AP components of the petalias (Fig. 3 and Table S3), the rightward AP frontal petalia asymmetry is more frequent in AMH (59%), and leftward AP asymmetry is predominant for the occipital petalia (73%). The RF/LO association for the AP component of the petalia is the most represented (44%). The results are similar in GA, although with lower values (57%R AP frontal, 57%L AP frontal). The RF/LO association for the AP component of the petalia is also the most frequent, but comprises only 35% of the specimens. Moreover, the asymmetry of the AP component of the occipital petalia is associated with a lateral component asymmetry toward the contra-lateral side in 74% of AMH and 77% of GA (Table S4). Finally, the lateral position of the poles, which is calculated relatively to the referential used to quantify the asymmetries, is significantly larger for the frontal poles in AMH compared to GA (p<0.001, means  =  7.4 and 5.5) and smaller (p<0.001, means  =  10.7 and 14.4) for the occipital poles, illustrating a different brain shape, in particular when considering the anterior part of the frontal lobes. Fossil hominins show intermediate values (6.6 and 13.8).

### Characteristics of fossil hominins

The heterogeneous composition of the fossil hominin sample does not allow a detailed statistical analysis of the variables distribution. Individual values for FA11 are larger in Sts 5 or KNM-WT 17000 (17.7) and in *Homo erectus s.l.* (N = 8, mean = 17.5) than in Neandertals (N = 4 for frontal petalias, 8 for occipital petalias, mean = 11.6). RF/LO association for the fronto-occipital AP petalial components is observed in 42% of the specimens (contra 44% in AMH and 35% in GA; Fig. 3) including KNM-WT 17000, KNM-ER 1813, 3883, OH 9, Ngandong 1, 7, 12 and Guattari (Table S1). Larger samples for each hominin species would be necessary to definitely confirm these results. However, the fossil hominin sample analysed here resembles AMH more than GA for the extent of asymmetries (FA11), their variation (Fig. 2 and Table S3) and their means (Fig. 2, multivariate test and Table S2).

## Discussion

Compared with qualitative assessments of petalial asymmetries, the quantification we here propose to characterize endocranial petalias is important for the study's repeatability. It also allows further exploration of the morphometric information contained in the analysed features. Moreover, because we used an external referential, our protocol is not influenced by endocranial asymmetries, as it is the case for most previous studies [35]. Finally, we here analysed “petalias” according to their original definition: the protrusions of one hemisphere beyond the other [31]. Unambiguous definition and consistent designation of the analysed features would be expected in brain asymmetry studies, together with protocols aimed at detecting and analysing the different main asymmetry patterns, including subtle departures from symmetry. In this context, functional significance of the various features [4]–[7], [19]–[21], [32]–[34] previously analysed in reference to the original works on the petalias [8], [9], [12], [31] is still under debate.

It first emerges from our analysis that the absolute size of the different petalial components within the various hominid samples is small (Table S2 and FA11). It then appears that the pattern of cerebral asymmetry is quite similar in AMH and GA with similar lateral orientation for most of the variables whose variation is related to fluctuating asymmetry (namely the AP, vertical and lateral components of the frontal petalias and the vertical component of the occipital petalias) and, more importantly, significant DA for AP and lateral components of the occipital petalias. Differences between AMH and GA reside in the orientation of the vertical component of the frontal and occipital petalias. We therefore observed a more lateralized distribution of the petalias and higher values for all the dimensions in AMH compared with GA (Figs. 2 and 3, Tables S2, S3, S4) for traits that are non significantly asymmetric. However, it appears from this study that GA exhibit significant endocranial asymmetry (DA) at the population level (Fig. 2) for two components of the petalias (the AP and lateral components of the occipital petalias). This pattern is mostly due to the features observed in *Pan paniscus* and *Pan troglodytes* [35] and is shared with AMH.

Neuroanatomical asymmetries of the human brain are thought to be related to lateralization of functions and we suggested elsewhere [35] that petalial pattern is not an epiphenomenon dependent on regional brain volumes. Large-scale anatomical asymmetries are however less easily interpretable than focal asymmetries as regards their relationships with brain function. It is therefore interesting that the large-scale combination of right frontal and left occipital protrusions was proposed to be characteristic of the hominin lineage [4]–[7] and hence became an argument for the supposed preponderance of right-handed individuals among fossil hominins since the appearance of stone flakes [6]. Based on analysis of “virtual” 3D models of the skull and endocranial cavity, the quantification of the AP component of endocranial petalias we here propose allows us to refine conclusions from qualitative descriptions of human brain asymmetries. Variations in the position of the frontal and occipital poles on the right and left hemispheres would be expected to show some degree of antisymmetry when population distribution is considered, but this is not the case. The observed pattern of variation among the samples is mostly related to fluctuating asymmetry. The distributions of AP and lateral components of the occipital petalia in AMH and GA, and probably in fossil hominins, however show significant directional asymmetry, the only form of asymmetry that is, at least partly, genetically heritable [40]. AMH, and probably fossil hominins, exhibit larger variation and larger relative dimensions for the quantified traits of petalial asymmetries than GA. This indicates that brain size expansion that occurred during hominin evolution and that strikingly differs between GA and hominins [6] was accompanied by a relative expansion of the degree of petalial asymmetries without modification of their pattern. This is especially interesting when considering previous observations on 403 *Macaca mulatta* endocranial casts [15], [17]. In these works, petalia, scored as non metric traits, represent frontal and occipital protrusions as well as the more lateral protrusion occurring between the orbitofrontal sulcus and the frontal pole. These studies revealed a significant occurrence of protrusions of the frontal pole in favour of the right side, whereas occipital petalia were not significant. Further studies [19] found no directional asymmetries in either Old or New World monkeys by using cerebral width as parameter but the size of the sample was probably too small. The frontal petalial pattern observed in macaque contrasts with the trend in favour of occipital petalia that we observed in GA and AMH. Falk et al. [15] and Cheverud et al. [17] further suggest that the finding of significant heritability for frontal petalia (protrusion) in macaques is consistent with a possible genetic component for cortical lateralization. The presence of DA revealed by our study indicates that the observed traits were probably inherited from at least the last common ancestor of chimpanzes, bonobos and humans. The mechanisms underlying the onset of petalial asymmetries in Old World monkeys and hominids are most probably similar, but led to different patterns of asymmetries. Interestingly, taken as a whole, the clade constituted by Old World monkeys, apes and humans appears to contrast with New World monkeys, where a completely different population-level left-frontal petalial pattern was found in a recent study of a population of 13 capuchin monkeys (*Cebus apella*) [41].

In general, anatomical asymmetries involve complex interactions between gender, handedness, and lateralization of functions [42]. There is for instance no significant gender effect on the well studied Planum Temporale asymmetry, neither in humans nor in great apes, in strong contrast with other asymmetries such as the one of the depth of the central sulcus, where an association was found between sulcal depth and handedness for male humans but not for females, suggesting gender differences in the cortical organization of human hand movements [43], [44]. These combinations differ between great apes and humans and no significant gender difference was found for central sulcal depth in chimpanzees [45].

Such complex interactions can be detected only when a sufficient number of subjects is available. In a voxel-based morphometric analysis of lobar asymmetry as well as Planum Temporale and hippocampus asymmetries performed on a large sample of 465 normal adult humans [32], no significant interaction between asymmetry and handedness and no main effect of handedness was detected. However, there was a significant main effect of gender on these brain asymmetries. This gender effect was observed in a previous study of cerebral widths [14] where the torque pattern of larger right anterior frontal and left parietal and occipital widths was found in right-handers together with a complex gender-related interaction where right-handed males and left-handed females showed a larger left parietal width. Other data [8], [33], [46], [47] lead to suggest a simpler gender-related difference in the degree of these shape asymmetries with a larger asymmetry in favour of the right frontal lateral extent in male than in female right-handers, whereas the asymmetry in favour of the left occipital lobe shows no gender differences in right-handers.

In contrast with these studies, we observed no gender impact for the petalial (protrusion) component asymmetries within our hominid samples. In concordance with our results, Falk et al. [15] observed no differences in protrusion asymmetries between the genders in their large sample of *Macaca mulatta*. It therefore appears to be possible that lobar asymmetries and protrusion asymmetries are uncoupled, at least when considering gender effect. This is important because the impact of each factor on anatomical asymmetries appears to be modified by the levels of the other ones [42].

In the absence of gender effect, the variation we observed in the degree of asymmetries in hominids may therefore be related to variation in handedness. In human, left occipital petalia is seen in 78% of right-handers, whereas left-handers are more often symmetric [48]. Using cerebral width as parameter, a right-frontal and left-occipital directional asymmetry was observed in great apes [19] but this sample was probably too small to detect any effect of gender or handedness. The lack of relationship between such lobar asymmetries and handedness is however in concordance with the above mentioned New World monkey study [41], where asymmetry in cerebral width was not related to handedness. It must here be mentioned that not all measures elicit population level handedness in non human primates, suggesting that hand preferences are task specific, with a sharp difference between the manipulative and communicative functions of the hands [49] and therefore different from what is observed in human populations. It moreover appears that in most studies of handedness in non human primates, the proportion of right-to-left individuals is ∼2∶1, a value that is much lower than the typical 8∶1 or 9∶1 ratio reported in human populations [23]. Therefore, although the population pattern of handedness could be similar, the difference in the degree of handedness is striking.

From our structural standpoint, the asymmetry pattern of the different components of the petalias is the same among hominids, but it is similarly striking to note that it is also a degree of asymmetry, here a degree of structural asymmetry, that appears to emerge as a relevant parameter. It is here worth mentioning that the degree of structural asymmetry could have possible functional significance [48], [50]. Such a relationship is for instance well illustrated in humans by the variations of the Planum Temporale asymmetry quotient among right-handed subjects, where the degree of leftward Planum Temporale asymmetry increases from right-handers with developmental dyslexia to normal right-handers, and is even much more pronounced in right-handed professional musicians with perfect pitch [51], [52]. Finally, although the available fossil hominin material is insufficient to pronounce on presence of asymmetries at the populational level, our results tend to indicate that the fossil hominin sample studied here show an intermediate pattern between GA and AMH for the degree of cerebral asymmetry, and therefore probably differences in their perceptual or motor skill performances compared to both groups [6].

Another potentially important observation is that the degree of petalial asymmetry is correlated with brain size across the sample and therefore that volumetric brain growth may be at the onset of structural and hence functional lateralization. This is in concordance with Ringo et al. [53]. Assuming that spatial clustering of interneuronal connections enhances cortical computation; these authors proposed that neural assemblies that are handling overlapping tasks are clustered together. This would be the essence of hemispheric specialization. It follows that hemispheric specialization would increase with brain size across mammals. Although we don't have enough species here to perform an analysis of possible scaling trends in the onset of our observed structural asymmetries, our results concerning the brain size related degree of structural asymmetry as a factor possibly shaping functional asymmetries, together with the proposal of Ringo et al. [53] are in concordance with the results of Smaers et al. [54]. These authors studied several parameters related to prefrontal cortex relative size in 19 primate species (including humans). This study reveals different scaling coefficients in the left versus right prefrontal hemisphere, and suggests a left hemisphere prefrontal hyperscaling with humans lying at the extreme of a left prefrontal ape specialization in relative white to grey matter volume. This also suggests again that cortical surface shape (or petalial pattern) and underlying cortical volume are decoupled [35].

Altogether, these results show that a specific pattern of protrusions of the frontal and occipital lobes appears to be a feature shared by all hominid primates, including extant African great apes, modern humans and fossil hominins. Assuming or not that GA are closer to the ancestral condition, they suggest, in contrast with previous studies [6], [7], [21], that the pattern of brain asymmetries is similar between great apes and hominins, leaving the gradient in the degree of asymmetry as the only relevant structural parameter. Therefore, although our study suggests that lateralized activities, some of them well probably distinctive to *Homo*, and cerebral lateralization are uncoupled, it also suggests that the quantitative differences in cerebral asymmetries lead to qualitative differences by permitting the arrival at thresholds and emergent functional properties [48]. In other words, change in magnitude of asymmetry is most probably accompanied by changes in hemispheric neuronal circuitry [30] significant enough to, at some point, induce changes in the functional capacity of the left and right brain systems.

## Materials and Methods

CT scans of the original specimens were used to obtain 3D models of the skull and of the endocranial cavity using customized settings for the precise reconstruction of fossilised or dry bone [35], [55]–[59] (Fig. 1A). The analyzed samples comprise 199 specimens, including 23 fossil hominins (Sts 5, KNM-WT 17000, KNM-ER 1813, KNM-ER 3733, 3883, OH 9, Broken Hill, LH 18, Ngandong 1, 7, 12, Ngawi, Sambungmacan 3, Liang Bua 1, Petralona, Gibraltar, Guattari, La Chapelle-aux-Saints 1, La Ferrassie 1, La Quina H5, Saccopastore 1 and Spy 1, 10), 21 fossil AMH (Afalou-Bou-Rhummel 2, 12, 28, 34, Cro-Magnon 1, 3; Mladeč 1, Nazlet Khater 2, Pataud, Rochereil, Skhul V, Song Terus, Taforalt XIc1, XIIc2, XVc2, XVc4, XVc5, XVIIc1, Téviec 8, 9, 16), 45 extant AMH and 110 African GA.

Thresholding procedures, three-dimensional volume rendering, and metrical analyses described below were undertaken with ArteCore 1 (NESPOS, available at https://www.nespos.org) or Avizo 6.1. Our objective was to determine the variation in location of the most protruding points on the right and left frontal and occipital lobes (*i.e.* the frontal and occipital poles; purple landmarks on Fig. 1A). We defined an external and independent referential based on anatomical points on the skull (glabella, inion and basion; red landmarks on Fig. 1A). In order to precisely describe the pattern of variation of endocranial petalias (protrusions of the frontal and occipital poles), we dissociated their antero-posterior (e.g., Fig. 1B), vertical (e.g., Fig. 1C) and lateral components (e.g., Fig. 1D). For that purpose, the coordinates of two lines were calculated from landmarks positioned on the external cranial surface. The first line (L1) passed through glabella and inion and the second line (L2) through basion and is orthogonal to the first. The most protruding points on the left and right frontal lobes and on the left and right occipital lobes were then projected orthogonally on L1. The distance on L1 between these projections corresponds to the antero-posterior component of the frontal and of the occipital petalia. These four endocranial landmarks were also orthogonally projected on L2. The distance on L2 between these projections corresponds to the vertical component of the petalia. Finally, these four endocranial landmarks were projected orthogonally on the plane defined by L1 and L2. The difference between the right and left lateral distance to the plane corresponds to the lateral component of the petalia. Different statistical procedures were used to analyze the recorded data and were conducted with PAST 2.09 software [60]. Grubbs' test statistic [61] was used to recognize the statistically significant outliers. Parametric (linear regression) and non-parametric (Spearman and Kendall coefficients of rank correlation) tests of association were used to test the relationship between the magnitude of the petalia and the size of the individuals. These parametric tests are preferred for this kind of analysis because they do not assume homogeneity of variance and are not influenced by the presence of a small number of outliers [36]. The values for kurtosis and skewness were calculated using Microsoft Excel. Kurtosis was compared to separate critical values for platy- and lepto-kurtosis ([36] table 5, values for equation 7). The statistical procedure to test for skewness and tables for critical values are detailed in Sokal and Rohlf [62]. Previous work has shown that “tests for skewness and kurtosis when taken together are probably the most useful way to detect departures from normality for metrical traits” ([63], p. 67). A sequential Bonferroni procedure was used for correction in multiple tests [37], [64]. Finally, Hotelling's T-squared, Mardia's multivariate and Box's M-statistical tests were also used in the course of multivariate analyses. Different potential sources of error were tested at the different steps of the analytical process, which was proved to be valid and reproducible (refer to [35] for a detailed description of the methodology).

## Supporting Information

Table S1 **Statistical tests** (Spearman coefficient of rank correlation, Kendall coefficient of rank correlation and linear correlation) of the relationships between the values of the different petalia components (|R-L|) and the endocranial volume (^3^√endocranial volume) for the complete hominid sample (including anatomically modern humans, great apes and fossil hominins); * indicates a p value <0.05, **<0.01, ***<0.001 after sequential Bonferroni procedure for correction for multiple tests.
(DOC)

Table S2 **Indices of asymmetry** **for anatomically modern humans (including fossil and extant specimens) and great apes; values for fossil hominins are also given but are only indicative as the heterogeneous composition of this sample does not allow detailed analysis of statistics and characteristics of the variables distribution.** (R-L): signed asymmetry is the mean difference between right and left side for each component of the petalia (or directional asymmetry) and is calculated for each sample, |R-L|: absolute asymmetry is the mean absolute value of the difference between the right and left side for each component of the petalia (or FA1) and is calculated for each sample, FA4a is calculated using the formula 0.798√var(R-L). All indices are size-corrected (*x_i_*/^3^√EV_i_*100).
(DOC)

Table S3 **Distribution of petalia components for anatomically modern humans (including fossil and extant specimens) and great apes, values for fossil hominins are also given but are indicative as the heterogeneous composition of this sample does not allow detailed analysis of statistics and characteristics of the variables distribution**.
(DOC)

Table S4 **Relationships of petalial components.** Relationships of antero-posterior petalial component with vertical and lateral petalial components for anatomically modern humans (including fossil and extant specimens) and great apes, values for fossil hominins are also given but are indicative as the heterogeneous composition of this sample does not allow detailed analysis of statistics and characteristics of the variables distribution.
(DOC)
