# Supplementary material for: Shared Pattern of Endocranial Shape Asymmetries among Great Apes, Anatomically Modern Humans, and Fossil Hominins
Source: PLoS One. 2012 Jan 5;7(1):e29581. doi: 10.1371/journal.pone.0029581 (PMC3252326; doi:10.1371/journal.pone.0029581)
Supplement: Table S2 — Indices of asymmetry for anatomically modern humans (including fossil and extant specimens) and great apes; values for fossil hominins are also given but are only indicative as the heterogeneous composition of this sample does not allow detailed analysis of statistics and characteristics of the variables distribution. (R-L): signed asymmetry is the mean difference between right and left side for each component of the petalia (or directional asymmetry) and is calculated for each sample, |R-L|: absolute asymmetry is the mean absolute value of the difference between the right and left side for each component of the petalia (or FA1) and is calculated for each sample, FA4a is calculated using the formula 0.798√var(R-L). All indices are size-corrected (xi/3√EVi*100). (DOC) [file pone.0029581.s002.doc]

**Table S2.**
