# Supplementary material for: Shared Pattern of Endocranial Shape Asymmetries among Great Apes, Anatomically Modern Humans, and Fossil Hominins
Source: PLoS One. 2012 Jan 5;7(1):e29581. doi: 10.1371/journal.pone.0029581 (PMC3252326; doi:10.1371/journal.pone.0029581)
Supplement: Table S3 — Distribution of petalia components for anatomically modern humans (including fossil and extant specimens) and great apes, values for fossil hominins are also given but are indicative as the heterogeneous composition of this sample does not allow detailed analysis of statistics and characteristics of the variables distribution. (DOC) [file pone.0029581.s003.doc]

**Table S3.**
